# Carbetocin versus oxytocin in prevention of postpartum hemorrhage after cesarean delivery in high-risk women. A systematic review and meta-analysis

**DOI:** 10.1007/s00404-025-08014-6

**Published:** 2025-04-16

**Authors:** Nour A. El-Goly, Ahmed Mohamed Maged, Wafaa M. Kamal, Osama Hosny, Doaa Turki, Nadia M. Helmy

**Affiliations:** 1 Faculty of Medicine, Cairo University, Cairo, Egypt; 2 Department of Obstetrics and Gynecology, Kasr Al-Ainy Hospital, Cairo University, Cairo, Egypt; 3 Departments of Physical Therapy for Woman’s Health, Faculty of Physical Therapy, Benha University, Benha, Egypt; 4 Department of Anaesthesia, Kasr Al-Ainy Hospital, Cairo University, Cairo, Egypt; 5 Department of Anaesthesia, National Cancer Institute, Cairo University, Cairo, Egypt

**Keywords:** Carbetocin, Oxytocin, Cesarean delivery, Blood loss, Postpartum hemorrhage

## Abstract

**Objectives:**

To assess the value of carbetocin in prevention of postpartum hemorrhage (PPH) after Cesarean delivery (CD).

**Search strategy:**

Screening of PubMed, Web Of Science, Scopus, register clinical trials registry and Google scholar from inception to December 2023. The keywords used included postpartum hemorrhage, intraoperative blood loss, postoperative blood loss, Cesarean delivery and their MeSH terms.

**Selection criteria:**

All RCTs that compared carbetocin to oxytocin in women undergoing CD with risk factor for PPH. Fourteen studies including 3068 participants. Thirteen were written in English and one in Polish.

**Data collection and analysis:**

The extracted data included location of the trial, number of centers involved in recruitment, the number of participants and their characteristics, details of the study groups and dose time and route of intervention and its comparator, primary and secondary outcome parameters and trial registration number and timing in relation to patients recruitment. The evaluated outcomes parameters included intraoperative and 1st 24 h post-operative blood loss, PPH, the hemoglobin changes after the procedure, the need for any additional uterotonic agents, surgical interventions or blood transfusion and drugs side effects.

**Main results:**

Blood loss during the 1st 24 h after CD was evaluated in 11 studies with 2497 participants and revealed a mean difference (MD) of −111.07 with 95% CI of [−189.34 and −32.80 (*P* = 0.005, *I*^2^ 97%). The hemoglobin changes after the operation was evaluated in 8 studies with 1646 participants and revealed a MD of −0.46 with 95% CI of −0.14 and −0.79 (*P* = 0.03, *I*^2^ 96%). The incidence of PPH > 500 ml was reported in 8 studies with 1787 participants and revealed an Odd Ratio (OR) of 0.52 with 95%CI of [0.36, 0.77] (*P* < 0.001, *I*^2^ 0%). The need for additional uterotonic agents was evaluated in 12 studies with 2663 participants and revealed an OR of 0.17 with 95% CI of 0.07 and 0.37 (*P* < 0.001, *I*^2^ 88%). The need for blood transfusion was evaluated in 10 studies with 2439 participants and revealed an OR of 0.27 with 95% CI of 0.12 and 0.57 (*P* < 0.001, *I*^2^ 20%). The need for additional interventions was evaluated in 3 studies with 1311 participants and revealed an OR of 0.67 with 95% CI of 0.28 and 1.60 (*P* = 0.37, I^2^ 59%).

**Conclusion:**

Carbetocin decreased the blood loss during the 1st 24 h after CD, post-operative hemoglobin drop, PPH the need for additional uterotonic agents and blood transfusion when compared to oxytocin.

**Supplementary Information:**

The online version contains supplementary material available at 10.1007/s00404-025-08014-6.

## Introduction

A million Cesarean deliveries (CDs) or more are carried on yearly in the United States being the most common surgical there [1]. World health organization reported an incidence of CD of 15% [2]. Although the rate of CD is increasing in both developed and developing countries, yet the incidence vary largely among different low income countries with a rate of 52% in Egypt and less than 5% in Somalia [3].

CD provides a lower risk of maternal pelvic floor and neonatal birth injuries but carries a higher maternal surgical risk during the current and future pregnancies and a higher risk for development of neonatal respiratory distress [4]. The maternal morbidity during CD is double that occurs during vaginal delivery [5] with higher rates of hemorrhage, infections, anesthetic, thromboembolic complications, and even death [6].

Postpartum haemorrhage (PPH) occurs in more than 14 million women and accounts for 70 000 maternal deaths yearly [7]. It is defined as more than 500 and 1000-mL blood loss during or within 24 h of VD and CD respectively [8]. Its risk is highest among low and middle income populations and the majority of maternal deaths in these countries are related to bleeding events as these counties lack the availability of high standard medical care [9].

The use of different uterotonic agents is recommended by WHO to prevent PPH in all deliveries [10]. The intravenous slow administration of 5 IU oxytocin is recommended by the Royal College of Obstetricians and Gynecologists after fetal extraction to ensure adequate contractions giving the advantages of rapid placental delivery and reduction of intraoperative and postpartum blood loss [11].

Oxytocin has many disadvantages. Its half life is short (4–10 min) so intravenous infusion is needed to achieve a prolonged action which necessitates continuous medical observation [12]. The other disadvantage is the requirement of a special storage and transport system with a 2–8 °C.

This is particularly important in limited resource areas especially in countries with hot and humid environments [13].

In 1997, an oxytocin analog with heat stability that binds oxytocin receptors and results in prolonged uterine contractions were developed. Carbetocin starts its action within 2 min and its action is maintained for 1 h after administration. The uterine contractions induced by carbetocin is one and half times more strong than that caused by oxytocin. Carbetocin presents an excellent alternative to the traditional oxytocin in less developed areas with limited storage and transport facilities [14].

## Objective

To compare the safety and efficacy of carbetocin and oxytocin in prevention of PPH after CD in high-risk women.

## Methods

The PRISMA guidelines of randomized controlled studies (RCTs) were followed in this systematic review. It was registered with CRD42023492407 number.

### Eligibility criteria, information sources, search strategy

The search databases included PubMed, Web Of Science, Scopus, register clinical trials registry and Google scholar from inception to December 2023. The key words used included carbetocin, postpartum hemorrhage, Intraoperative blood loss, Postoperative blood loss, Cesarean delivery and their MeSH terms. All related clinical trials and reviews reference lists were checked for possible study inclusions.

### Study selection

Study selection based on PICO format. Population included women underwent CD with high risk for development of postpartum hemorrhage. Intervention women received carbetocin injection. Comparator: studies comparing carbetocin to oxytocin alone or when combined with another uterotonic drugs as misoprostol. Outcomes: blood loss. Types of included studies: only RCTs without any language limitations. Cohort, case control studies, case series, reviews and editorial opinion were excluded. All doses and routes of administration of both oxytocin and carbetocin were included.

The database search, assessment of all articles and evaluation for inclusion or exclusion from the review were done by two authors independently. Any disagreement between them was discussed with other authors.

After selection of the included studies, the data were extracted by two authors independently and any needed clarifications were obtained by contacting the corresponding author and other co-authors. The extracted data included location of the trial, number of centers involved in recruitment, the number of participants and their characteristics, details of the study groups and dose time and route of intervention and its comparator, primary and secondary outcome parameters and trial registration number and timing in relation to patients recruitment.

The evaluated outcomes parameters included intraoperative and 1st 24 h post-operative blood loss, PPH, the hemoglobin changes after the procedure, the need for any additional uterotonic agents, surgical interventions or blood transfusion and drugs side effects.

The Cochrane Handbook of Systematic Reviews recommendation for evaluation of RCTs were followed. Evaluated items included the generation of random sequence, allocation concealment, blinding of participants and outcome assessors, incomplete and selective data reporting beside the evaluation of other biases. The quality of evidence was evaluated using GRADE analysis which.

Two authors (SA and WSR) assessed the quality of the individual studies following recommendations that include the number of the studies, their risk of bias, outcome inconsistency, indirectness of data, sample size and width of CI and publication bias.

### Statistical analysis

The mean differences and OR and their 95% confidence interval (CI) were used to calculate the effect estimate of continuous and dichotomous data, respectively. The fixed or random effect was used according to studies heterogeneity. The heterogeneity was evaluated through assessed by Cochran’s Q test and *I*^2^ statistic and a significant hetero1geneity was considered when *I*^2^ statistic reached more than 40%. A significant effect was considered when the P value was below 0.05. All statistical calculations were done using the Review Manager (RevMan) version 5.4.1 (The Nordic Cochrane Centre, Cochrane Collaboration, 2020, Copenhagen, Denmark).

## Results

### Study selection, study characteristics

**Fig. 1:**
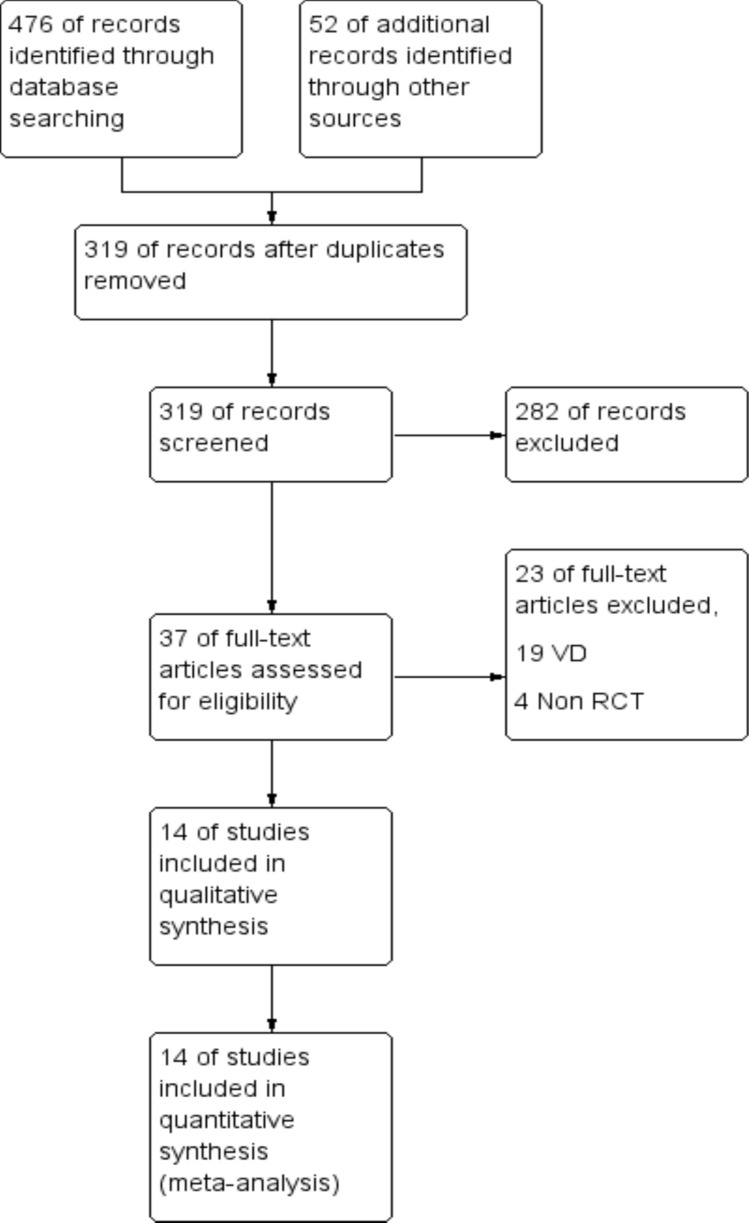
PRISMA flow chart

The PRISMA flow chart describing the search results is shown in Fig. 1

Fourteen studies including 3068 participants (1519 subjected to carbetocin and 1398 subjected to oxytocin, 151 were subjected to B Lynch technique) were included in our meta-analysis [15–28]. Thirteen were published in English and one in Polish language [23].

All the studies were 2 arm except Mohamed et al. [25] was 3 arm study. Carbetocin was compared to oxytocin alone in one studies and compared to combined oxytocin and misoprostol in one study [20]. CD in the included studies was elective in seven studies, emergency in two studies and a mixture of both elective and emergency in five studies. CD were conducted under spinal anesthesia in six studies, under general anesthesia in two studies while in six studies the type of anesthesia was not specified. The dose of oxytocin was 5 IU in 1 study [16], 10 IU in 4 studies [17, 22, 23, 28], 20 IU in five studies [19–21, 25, 26], 30 IU in three studies [18, 24, 27] and 40IU in one study [15]. The risk factor for PPH was specified as hypertensive disorder in three studies [15, 16, 22], Twin pregnancy in one study [21], obesity in one study [19] and general non-specified risk factor in nine studies.

Eleven studies were conducted in a single center and three studies were conducted in two centers [19, 25, 27]. Seven studies were conducted in Egypt, 2 were conducted in Italy, one study in each of the following countries Bangladesh, China, Iran, Poland and Thailand. Among the included studies only five trials were prospectively registered.

The characteristics of the included studies are presented in Table 1.

**Table 1:** Characteristics of the included studies

Study	Settings	Sample size	Participants	Interventions	Outcomes	Other
Al-Anwar 2022	Single center Egypt	120 randomized 120 analyzed	Inclusion criteria: Preeclampsia CS elective or emergency GA > 28 weeks Exclusion criteria: HELLP syndrome. Multiple pregnancy Placenta previa or abruption. Women with significant heart disease, liver, renal disease Coagulopathy. Allergy to carbetocin or oxytocin	Study group (n = 60) received 100 μg Carbetocin diluted in 10 mL 0.9 saline IV over 10 min I/V after fetal delivery Control group (n = 60) received 40 IU oxytocin diluted in 100 mL of 0.9% saline IV over 4 h after fetal delivery	Primary outcome: PPH that require additional uterotonics Secondary outcomes: The need and amount of blood transfusion, hemoglobin and hematocrit changes, vital signs, hospital stay, maternal complications (fever, DIC, infection, ICU admission), The need for additional interventions adverse effects	Not registered
Bahr 2023	Single center Egypt	80 randomized 80 analyzed	Inclusion criteria: Mild Preeclampsia CS elective under spinal anesthesia GA Term Exclusion criteria: Regional anesthesia refusal or contraindications (e.g., coagulopathy), placenta previa, or abruption, and multiple gestation, pregnancy-related complications as GDM diseases thyroid disorders, cardiovascular, renal diseases or diabetes	Study group (n = 40) received 100-μg Carbetocin diluted in 10 mL 0.9 saline IV over 10 s Control group (n = 40) received 5 IU oxytocin diluted in 10 mL of 0.9% saline IV over 10 s	Primary outcome Blood pressure (mean, systolic and diastolic) Secondary outcomes Heart rate O2 saturation Myocardial ischemia Hypotension Intraoperative ephedrine use The need for additional uterotonics PPH Blood loss Postoperative hemoglobin	Prospective registration (PACTR201909623653208)
Borruto 2009	Single center Italy	104 randomized 104 analyzed	Inclusion criteria: Singleton pregnancy with one or more risk factors for PPH GA > 36 weeks CS elective and selective Exclusion criteria: Toxemia, eclampsia, and epilepsy	Study group (n = 52) received 100 μg Carbetocin IV immediately after placental delivery Control group (n = 52) Received 10 IU oxytocin IV infusion over 2 h	Primary outcome The need for additional uterotonics Secondary outcomes: Safety and ability to maintain adequate uterine tone Incidence and severity of PPH	Not registered
De Bonis 2012	Single center Italy	110 randomized 110 analyzed	Inclusion criteria: One or more risk factors for PPH GA > 37 weeks CS elective Exclusion criteria: preeclampsia, eclampsia, cardiovascular, renal, liver diseases and epilepsy General anesthesia	Study group (n = 55) received 100-ug carbetocin diluted in 10-ml 0.9% saline IV over 30–60 s after fetal and before placenta delivery Control group (n = 55) received 10-IU oxytocin diluted in 10-ml 0.9% saline IV over 30–60 s after fetal and before placenta delivery followed by 20 IU IV infusion over 24 h	Vital signs Uterine involution Lochia Hemoglobin changes Pain VAS score The need for analgesics The need for diuretics Drugs Side effects	Not registered
El-Behery 2016	2 centers Egypt	180 randomized 180 analyzed	Inclusion criteria: Nullipara Singleton fetus GA ≥ 37 weeks BMI > 30 kg/m^2^ CS emergency Exclusion criteria: multigravida malpresentation VD Elective CS	Study group (n = 90) received 100-ug carbetocin diluted in 10-ml ringers lactate IV over 2 min after fetal and before placenta delivery and 4ml Ringer’s lactatein in 1000 ml of Ringer’s lactate IV infusion (125 ml/h) Control group (n = 90) received 20 IU in 1000 ml of Ringer’s lactate IV infusion (125 ml/h) and 11-ml ringer lactate IV over 2 min	Primary outcome Major PPH Secondary outcomes Hemodynamic effects The need for blood transfusion Hemoglobin and hematocrit changes The need for additional uterotonics Uterine tone Drugs side effects	Not registered
Elgafor el Sharkwy 2013	Single center Egypt	380 randomized 380 analyzed	Inclusion criteria: CS elective One or more risk factors for PPH Exclusion criteria: Coagulopathy General anesthesia Hypertension, cardiovascular diseases Non atonic PPH Allergy to carbetocin	Study group (n = 190) Received 400-ug sublingual misoprostol after spinal anesthesia and few minutes before skin incision plus 20-IU oxytocin in 500-mL solution IV infusion over 15 min after fetal delivery Control group (n = 190) received 100-ug carbetocin diluted in 10-ml normal saline IV over 30 60 s after fetal delivery	Primary outcome The need for additional uterotonics Secondary outcomes Hemoglobin changes Amount of blood loss The need for blood transfusion, Drug side effects	Not registered
Fahmy 2016	Single center Egypt	60 randomized 60 analyzed	Inclusion criteria Twin pregnancy ASA physical status I Age 28–36 years CS elective Exclusion criteria Higher order pregnancy Preoperative hemoglobin < 9.5 gm% Diseases as hypertension, preeclampsia, heart, lung, liver, or kidney Bleeding disorder such as hemophilia anticoagulants therapy Allergy to carbetocin and / or oxytocin	Study group (n = 30) received 100-ug carbetocin in 10-ml saline slowly IV over 1 min after fetal delivery Control group (n = 30) received 20 IU oxytocin in 10 ml of saline solution slowly IV over 1 min after fetal delivery	Hemodynamic parameters Uterine contraction score The need for additional uterotonics Blood loss	Not registered
Ibrahim 2020	Single center Egypt	160 randomized 160 analyzed	Inclusion criteria Age 18–35 years Singleton pregnany CS elective GA ≥ 37 weeks Hypertension disorder in pregnancy Exclusion criteria Other risk factors for PPH as placenta previa or uterine fibroid Previous thromboembolic events Diseases as heart, liver or kidney CS on request General anesthesia	Study group (n = 80) received 100-μg carbetocin diluted in 10 ml of Ringer lactate IV over 2 min Control group (n = 80) received 10 IU oxytocin in 100 ringer lactate IV infusion (125 ml/h) after fetal and before placental delivery	Primary outcome Major PPH Secondary outcomes Vital signs The need for blood transfusion Hemoglobin and hematocrit changes The need for additional uterotonics Uterine tone	Prospective registration PACTR201909807831604
Jagielska 2015 Article in Polish	Single center Poland	190 randomized 190 analyzed	Inclusion criteria Women with one or more risk factors for PPH GA 25–41 weeks	Study group (n = 130) received 100 μg of IV carbetocin after cord clamping Control group (n = 60) eceived 10 units of oxytocin i.v. after cord clamping	Primary outcome Hemoglobin changes Secondary outcomes Blood loss PPH	Not registered
Kang 2022	Single center China	852 randomized 827 analyzed	Inclusion criteria Women with one or more risk factor for PPH CS elective GA term Exclusion criteria Age < 18 years Multiple pregnancy placenta praevia or suspected accreta, Hypertension, heart, liver, kidney or endocrine diseases systematic disease (except diabetes), coagulopathy Allergy to carbetocin or oxytocin	Study group (n = 439) received 100-ug carbetocin IV over 1 min after fetal delivery Control group (n = 388) received 10 IU oxytocin intrauterine followed by 20 IU oxytocin in 500-mL 5% glucose IV drip over 1 h after fetal delivery	Primary outcome The need for additional uterotonics Secondary outcomes Blood loss (intraoperative, within 2 and 24 h after CS) PPH The need for blood transfusion The need for additional interventions The need for hemostatics	Prospective registration ChiCTR1800015613
Mohamed 2016	2 center Egypt	453 randomized 453 analyzed	Inclusion criteria Age 21–35 years GA 35–40 weeks Parity 0–4 BMI 20–35 kg/m2 One or more risk factors for PPH Exclusion criteria Medical disorders with pregnancy (hypertension,diabetes, cardiovascular diseases, epilepsy, migraine and bronchial asthma	Group 1 (n = 151) underwent prophylactic B-Lynch suturing after closure of the uterus Group 2 (n = 151) received 100 ug of carbetocin IV after closure of the uterus Group 3 (n = 151) received 20 IU of oxytocin infusion on 1000-ml saline at a rate of 250 ml/h after closure of the uterus	Primary outcome Intraoperative blood loss Major PPH The need for additional uterotonics Secondary outcomes Hemoglobin changes The need for blood transfusion Hospital stay Adverse effects	Not registered
Sudjai 2022	Single center Thailand	120 randomized 120 analyzed	Inclusion criteria Singleton pregnancy Age ≥ 18 years CS elective or emergency GA > 34 weeks One or more risk factors for PPH Exclusion criteria multiple gestations, placenta previa, abruption or accreta Diseases as hypertension, preeclampsia, cardiac, renal diseases or coagulopathy	Study group (n = 60) Carbetocin 100 µg + ringer lactate solution (RLS) 10 mL in syringe 20 mL IV over 30—60 s and followed with RLS 1000 mL plus RLS 4 mL IV infusion over 8 h (120 mL/hour) Control group (n = 60) RLS 11 mL in syringe 20 mL IV over 30 t- 60 s and followed with RLS 1000 mL plus oxytocin 20 IU IV infusion over 8 h (120 mL/hour)	Primary outcome The need for additional uterotonics Secondary outcomes Blood loss Hemoglobin changes Hospital stay Drugs side effects	Prospective registration NCT04089176
Taheripanah 2018	2 centers Iran	220 randomized 220 analyzed	Inclusion criteria One or more risk factor for PPH GA > 37 weeks CS emergency Exclusion criteria Refusal to participate Allergy to carbetocin or oxytocin Multiple pregnancy preeclampsia, heart or kidney diseases Major therapeutic side effects	Study group (n = 110) Received 100-ug carbetocin IV Control group (n = 110) received 30IU oxytocin IV infusion during 2 h after delivery of placenta	Primary outcome PPH The need for additional uterotonics Secondary outcomes Blood loss Hemoglobin changes Vital signs Drugs adverse effects	Prospective registration NCT02079558
Yesmin 2022	Single center Bangladish	64 randomized 64 analyzed	Inclusion criteria GA ≥ 37 weeks CS elective or emergency under spinal anesthesia One or more risk factors for PPH Exclusion criteria Hypertension or preeclampsia Diseases as heart, liver, kidney diseases or epilepsy Allergy to carbetocin or oxytocin Cs under general anesthesia	Study group (n = 32) Received 100-ug carbetocin IV immediately after fetal delivery Control group (n = 32) Received 10 IU oxytocin IV immediately after fetal delivery	Vital signs Blood loss Hemoglobin changes Uterine tone Uterine position Urine output The need for additional uterotonics PPH The need for blood transfusion Drugs side effects Costs	Not registered

**Fig. 2:**
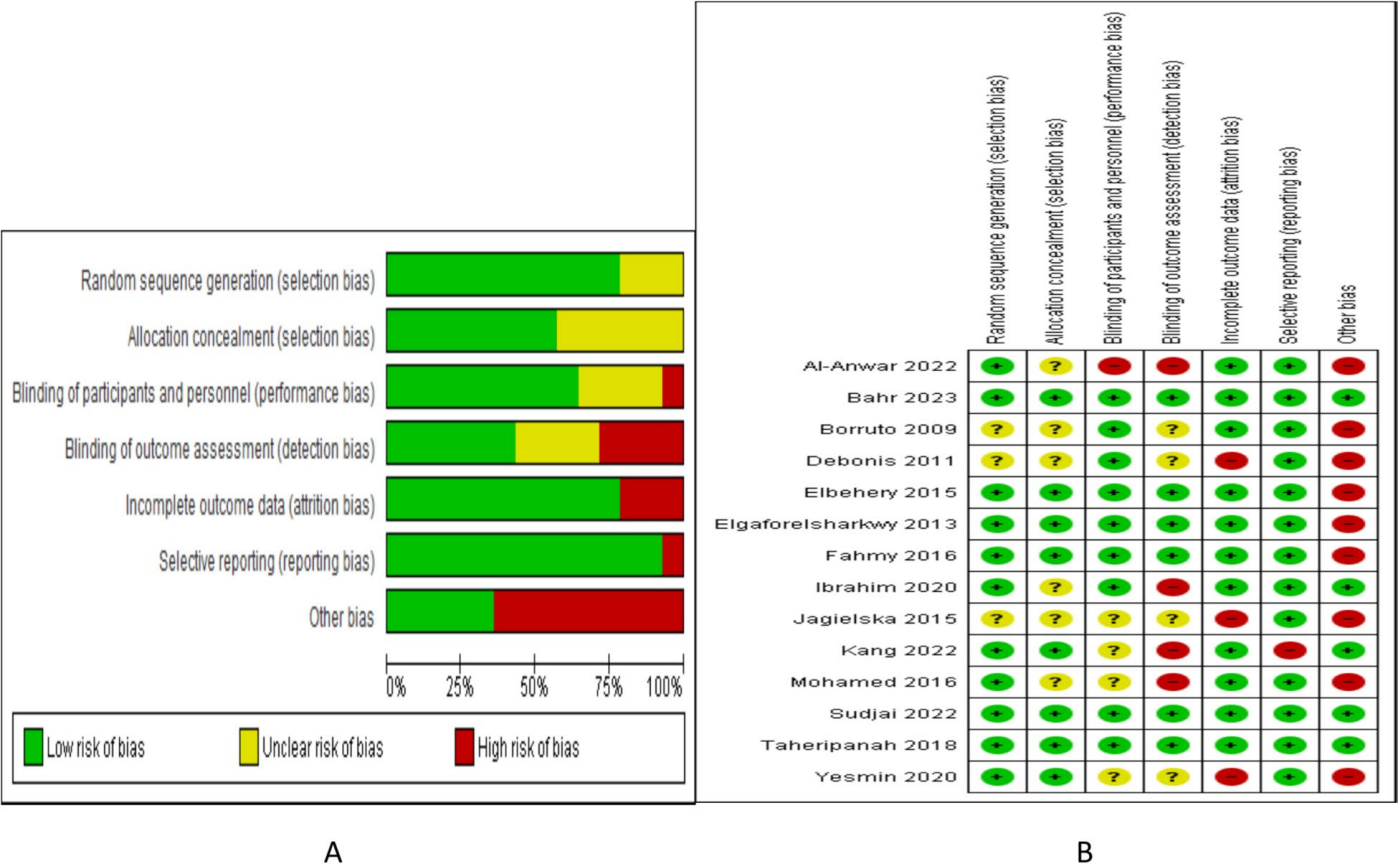
Risk of bias **A** graph **B** summary

Figure 2 illustrate the risk of bias graph and summary and Table 2 describe the GRADE quality of evidence.

**Table 2:** GRADE quality of evidence

Outcome	No studies	Risk of bias	Inconsistency	Indirectness	Imprecision		Publication bias	Quality
					Sample size	Wide CI		
Blood loss	11	N	N	N	2497	N	N	High
Hemoglobin changes	8	N	S	N	1646	N	N	Moderate
Postpartum hemorrhage	8	N	N	N	1787	N	N	High
The need for additional uterotonic agents	12	N	N	N	2663	N	N	High
The need for blood transfusion	10	N	N	N	2439	N	N	High
The need for additional interventions	3	N	S	N	1311	S	N	Low
Nausea and/or vomiting	6	N	S	N	1124	S	N	Low
Headache	5	N	N	N	1004	N	N	High
Abdominal pain	3	N	S	N	344	S	N	Very low
Fever	3	N	S	N	680	S	N	Very low
Hypotension	2	N	S	N	500	S	N	Very low

### Synthesis of results

**Fig. 3:**
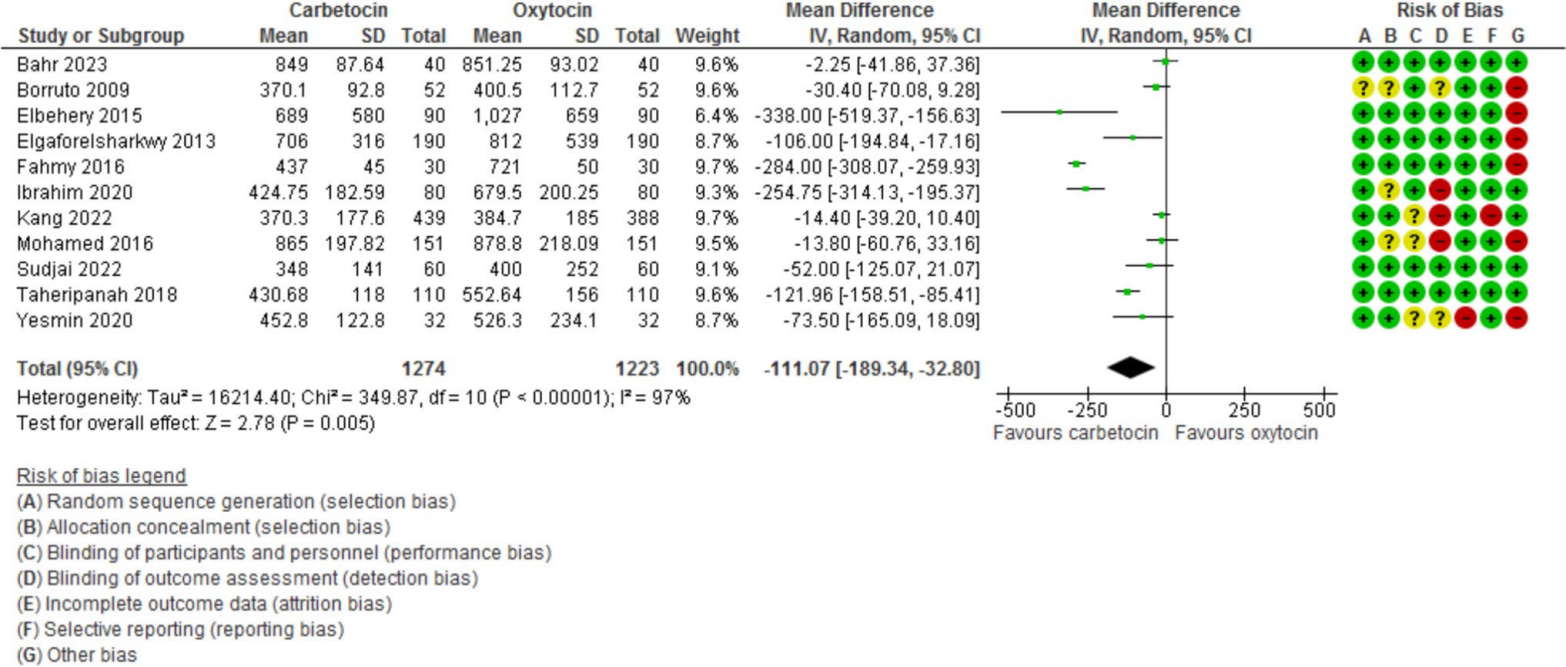
Blood loss within 24 h after CD

**Fig. 4:**
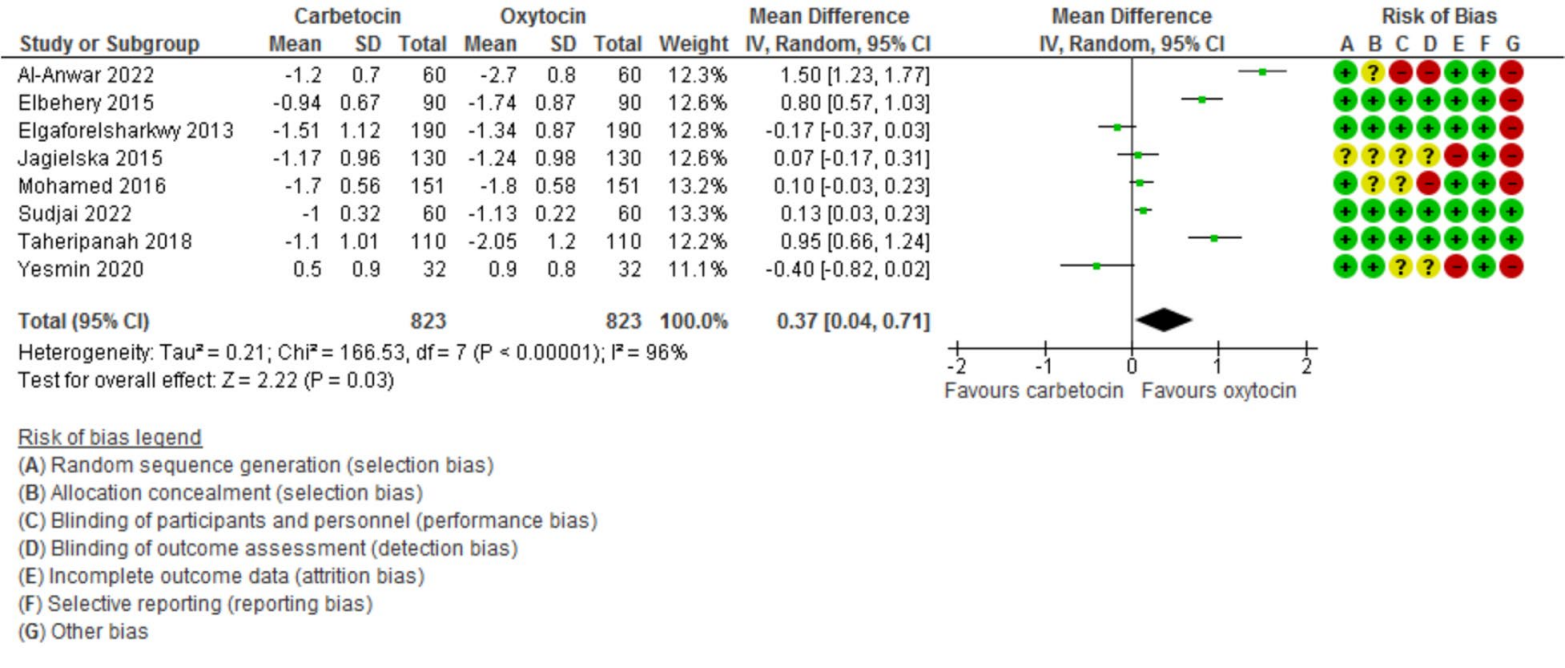
The difference between preoperative and post-operative Hemoglobin

**Fig. 5:**
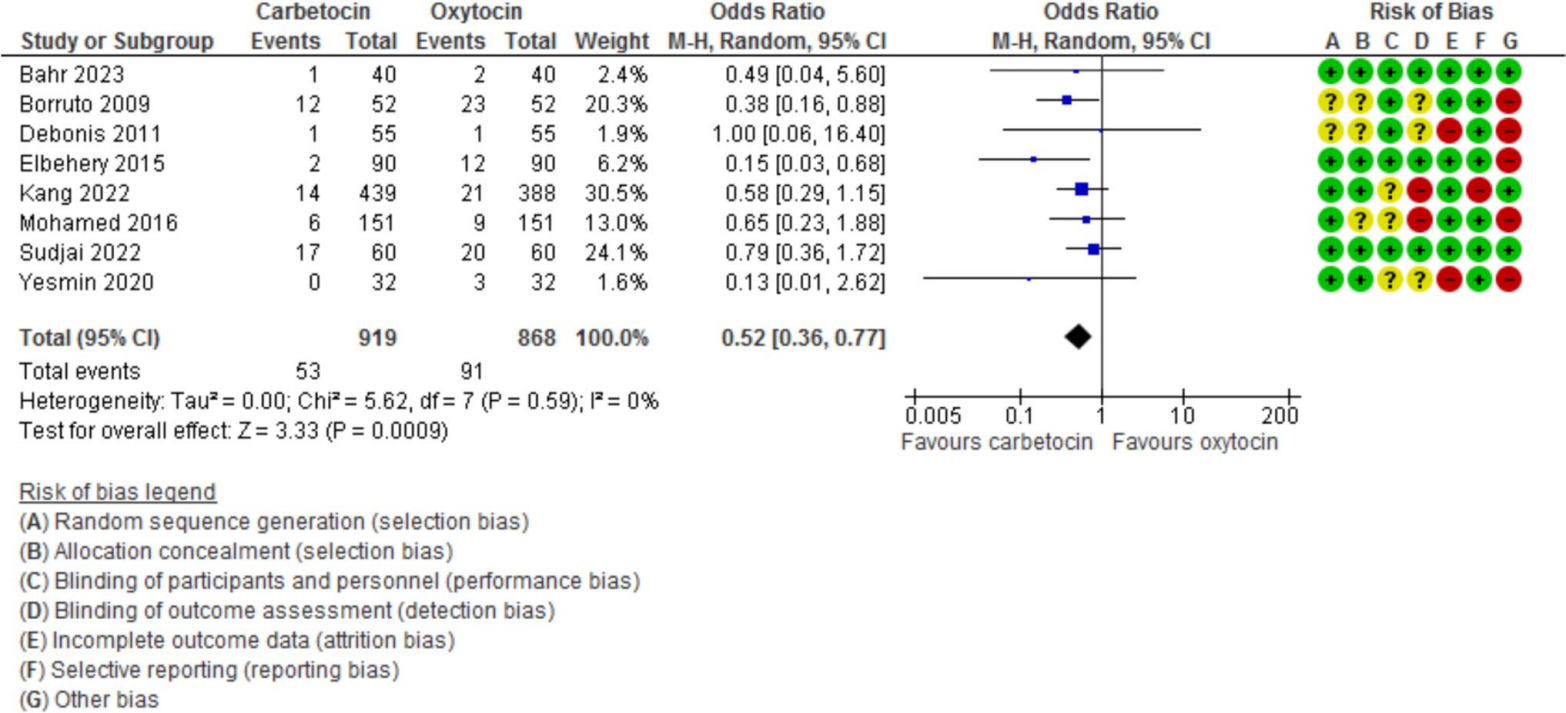
the incidence of PPH

**Fig. 6:**
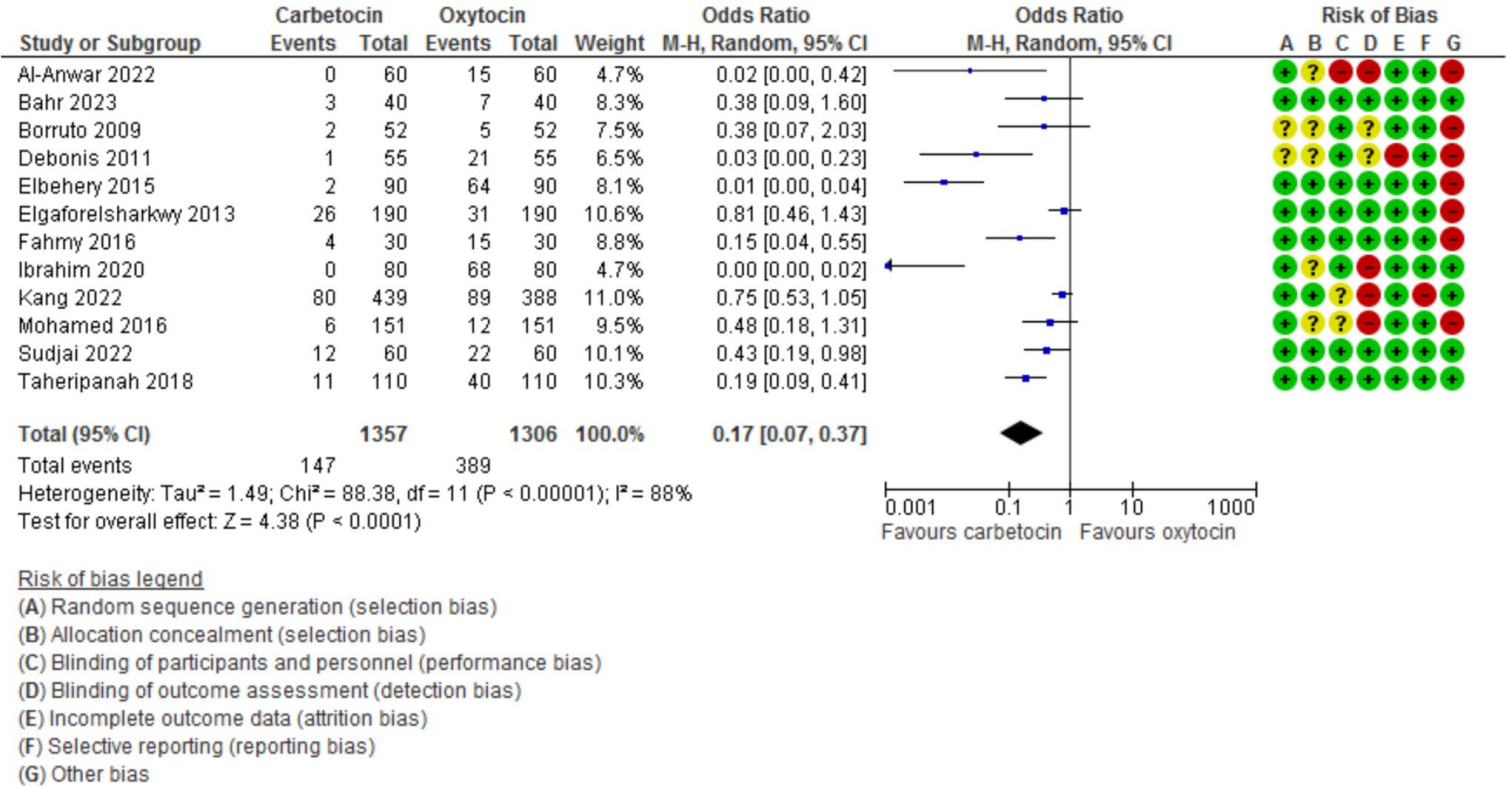
The need additional uterotonic agents

**Fig. 7:**
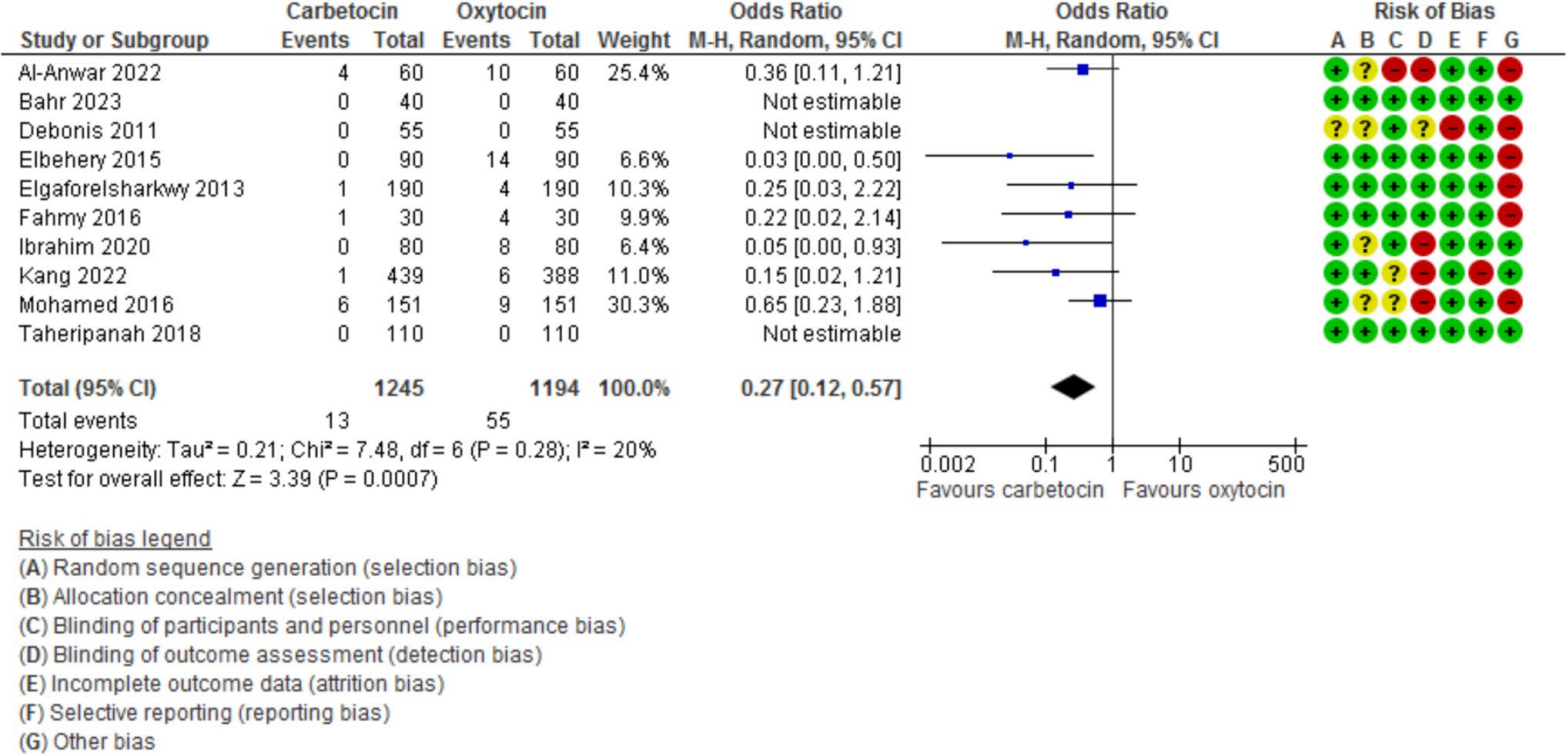
The need for blood transfusion

**Fig. 8:**
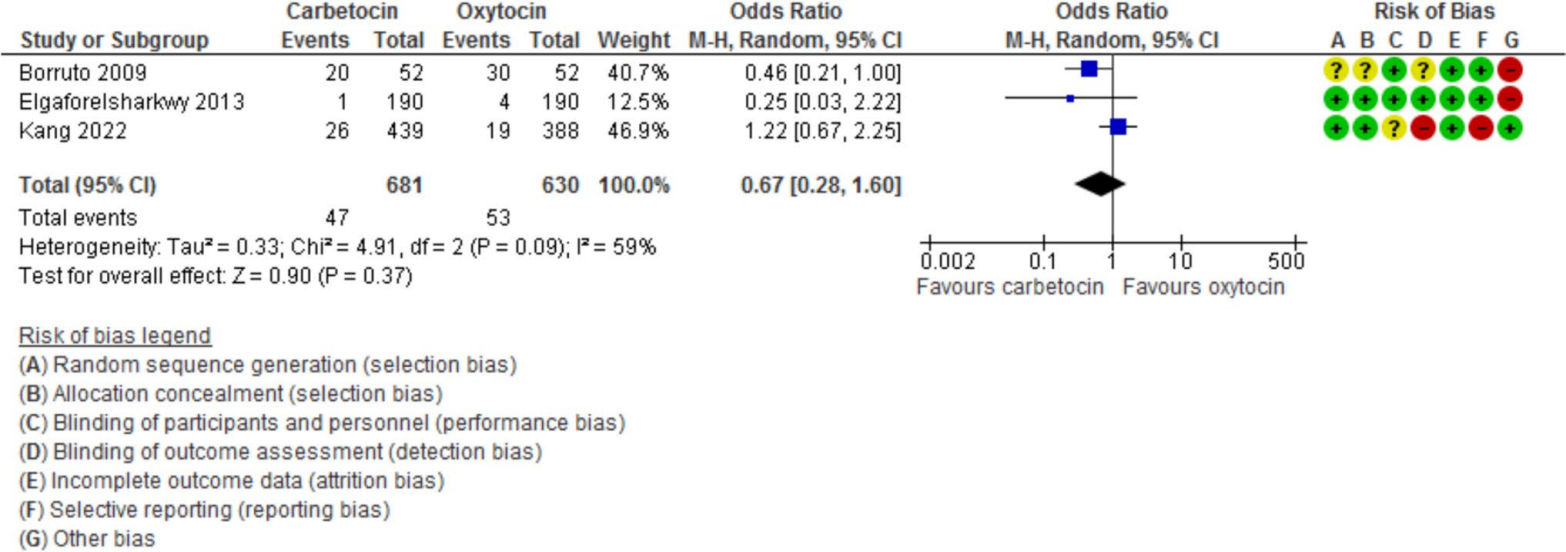
The need for additional interventions

Blood loss during the 1st 24 h after CD was evaluated in 11 studies with 2497 participants and revealed a MD of −111.07 with 95% CI of [−189.34 and −32.80 (*P* = 0.005, *I*^2^ 97%) (Fig. 3). The hemoglobin changes after the operation was evaluated in 8 studies with 1646 participants and revealed a MD of −0.46 with 95% CI of −0.14 and −0.79 (*P* = 0.03, *I*^2^ 96%) (Fig. 4). The incidence of PPH > 500 ml was reported in 8 studies with 1787 participants and revealed an OR of 0.52 with 95%CI of [0.36, 0.77] (*P* < 0.001, *I*^2^ 0%) (Fig. 5). The need for additional uterotonic agents was evaluated in 12 studies with 2663 participants and revealed an OR of 0.17 with 95% CI of 0.07 and 0.37 (*P* < 0.001, *I*^2^ 88%) (Fig. 6). The need for blood transfusion was evaluated in 10 studies with 2439 participants and revealed an OR of 0.27 with 95% CI of 0.12 and 0.57 (*P* < 0.001, *I*^2^ 20%) (Fig. 7). The need for additional interventions was evaluated in three studies with 1311 participants and revealed an OR of 0.67 with 95% CI of 0.28 and 1.60 (*P* = 0.37, *I*^2^ 59%) (Fig. 8).

**Fig. 9:**
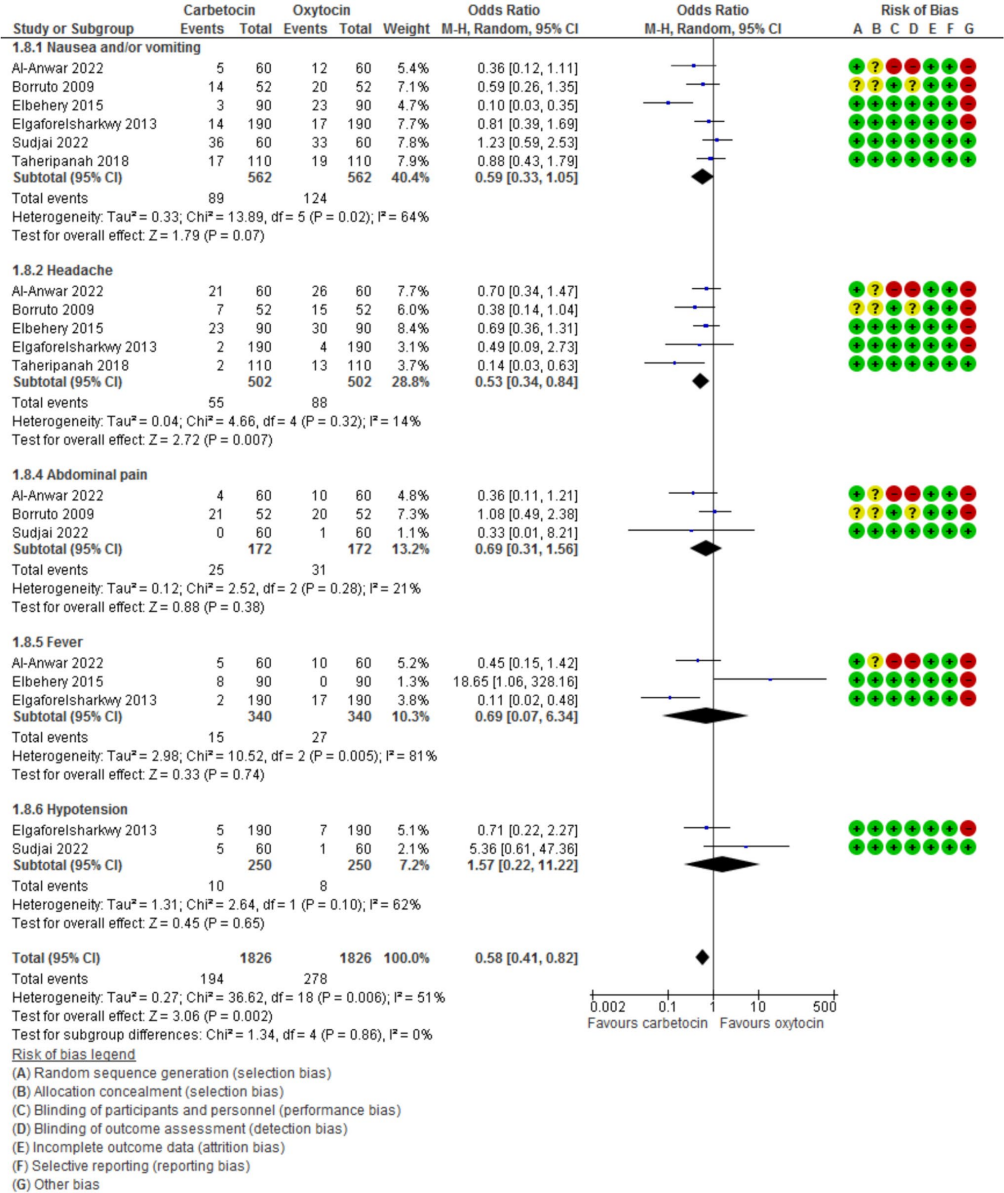
Side effects

Evaluation of adverse effects (Fig. 9) revealed that nausea and/or vomiting was evaluated in six studies with 1124 participants and showed an OR of 0.59 with (0.33, 1.05) 95% CI (*P* = 0.07, *I*^2^ 64%). Headache was evaluated in five studies with 1004 participants and showed an OR of 0.53 with (0.34, 0.84) 95% CI (*P* = 0.007, *I*^2^ 14%). Abdominal pain was evaluated in three studies with 344 participants and showed an OR of 0.69 with (0.31, 1.56) 95% CI (*P* = 0.38, *I*^2^ 21%). Fever was evaluated in 3 studies with 680 participants and showed an OR of 0.69 with (0.07, 6.34) 95% CI (*P* = 0.74, *I*^2^ 81%). Hypotension was evaluated in 2 studies with 500 participants and showed an OR of 1.57 with (0.22, 11.22) 95% CI (*P* = 0.65, *I*^2^ 51%).

Subgroup analysis according to the dose of oxytocin, the type of anesthesia, the defined risk for PPH and the type of CD for all study outcomes are described in Table 3.

**Table 3:** Subgroup analysis of the study outcomes

	Outcome	Subgroup	No of studies	No of participants	Effect estimate OR [95% CI]	P value	Heterogeneity *I*^2^
Dose of oxytocin	Blood loss	5 IU	1	80	−2.25 [−41.86, 37.36]	0.91	NA
		10 IU	3	328	−119.70 [−271.59, 32.20]	0.12	95%
		20 IU	5	1042	−151.67 [−299.29, −4.05]	0.04	97%
		30 IU	2	1047	−67.31 [−172.70, 38.09]	0.21	96%
		Total	11	2497	−111.07 [−189.34, −32.80]	0.005	97%
	Hemoglobin changes	10 IU	2	324	0.19 [−0.12, 0.50]	0.23	45%
		20 IU	4	982	0.21 [−0.08, 0.49]	0.15	93%
		30 IU	1	220	0.95 [0.66, 1.24]	< 0.001	NA
		40 IU	1	120	1.50 [1.23, 1.77]	< 0.001	NA
		Total	8	1646	0.46 [0.14, 0.79]	< 0.001	96%
	PPH	5 IU	1	80	0.49 [0.04, 5.60]	0.56	NA
		10 IU	2	168	0.35 [0.15, 0.79]	0.01	0%
		20 IU	3	602	0.51 [0.22, 1.21]	0.13	48%
		30 IU	2	937	0.59 [0.30, 1.16]	0.13	0%
		Total	8	1787	0.52 [0.36, 0.77]	< 0.001	0%
	The need for additional uterotonic agents	5 IU	1	80	0.38 [0.09, 1.60]	0.19	NA
		10 IU	2	264	0.02 [0.00, 11.82]	0.24	93%
		20 IU	5	1042	0.21 [0.06, 0.76]	0.02	89%
		30 IU	3	1157	0.22 [0.05, 0.93]	0.04	90%
		40 IU	1	120	0.02 [0.00, 0.42]	0.01	NA
		Total	12	2663	0.17 [0.07, 0.37]	< 0.001	88%
	The need for blood transfusion	5 IU	1	80	NE	NE	NA
		10 IU	1	160	0.05 [0.00, 0.93]	0.04	NA
		20 IU	4	922	0.26 [0.07, 0.96]	0.04	43%
		30 IU	3	1157	0.15 [0.02, 1.21]	0.07	NA
		40 IU	1	120	0.36 [0.11, 1.21]	0.10	NA
		Total	10	2439	0.27 [0.12, 0.57]	< 0.001	20%
Type of anesthesia	Blood loss	Spinal	5	1511	−87.95 [−175.21, −0.69]	0.05	93%
		General	2	362	−149.68 [−414.47, 115.10]	0.27	99%
		Non-specified	5	624	−100.23 [−175.80, −24.66]	0.009	85%
	Hemoglobin changes	Spinal	2	444	0.08 [−0.47, 0.64]	0.77	83%
		General	1	302	0.10 [−0.03, 0.23]	0.13	NA
		Non-specified	5	900	0.68 [0.17, 1.20]	0.009	97%
	PPH	Spinal	4	1081	0.55 [0.29, 1.03]	0.06	0%
		General	1	302	0.65 [0.23, 1.88]	0.43	NA
		Non-specified	3	404	0.42 [0.18, 0.97]	0.04	53%
	The need for additional uterotonics	Spinal	5	1557	0.19 [0.05, 0.68]	0.01	90%
		General	2	362	0.29 [0.10, 0.88]	0.03	47%
		Non-specified	5	744	0.11 [0.03, 0.44]	0.002	84%
	The need for blood transfusion	Spinal	5	1557	0.14 [0.04, 0.55]	0.005	0%
		General	2	362	0.54 [0.21, 1.40]	0.21	0%
		Non-specified	3	520	0.13 [0.01, 1.89]	0.14	68%
Risk factors	Blood loss	Hypertensive disorders	2	240	−127.49 [−374.93, 119.95]	0.31	98%
		Twins	1	60	−284.00 [−308.07, −259.93]	< 0.001	NA
		Obesity	1	180	−338.00 [−519.37, −156.63]	< 0.001	NA
		General	7	2017	−54.74 [−93.50, −15.98]	0.006	78%
	Hemoglobin changes	Hypertensive disorders	1	120	1.50 [1.23, 1.77]	< 0.001	NA
		Obesity	1	180	0.80 [0.57, 1.03]	< 0.001	NA
		General	6	1346	0.22 [−0.00, 0.43]	0.05	88%
	PPH	Hypertensive disorders	1	80	0.49 [0.04, 5.60]	0.56	NA
		Obesity	1	180	0.15 [0.03, 0.68]	0.01	NA
		General	6	1527	0.57 [0.38, 0.85]	0.006	0%
	The need for additional uterotonics	Hypertensive disorders	3	360	0.02 [0.00, 1.24]	0.06	88%
		Twins	1	60	0.15 [0.04, 0.55]	0.004	NA
		Obesity	1	180	0.01 [0.00, 0.04]	< 0.001	NA
		General	7	2063	0.41 [0.23, 0.73]	0.002	72%
	The need for blood transfusion	Hypertensive disorders	3	360	0.21 [0.04, 1.23]	0.08	37%
		Twins	1	60	0.22 [0.02, 2.14]	0.19	NA
		Obesity	1	180	0.03 [0.00, 0.50]	0.01	NA
		General	5	1839	0.44 [0.18, 1.04]	0.06	0%
Type of CD	Blood loss	Elective	6	1809	−112.36 [−235.99, 11.27]	0.07	98%
		Emergency	2	400	−210.97 [−419.38, −2.56]	0.05	81%
		Both	3	288	−40.15 [−72.74, −7.56]	0.02	0%
	Hemoglobin changes	Elective	2	682	−0.02 [−0.29, 0.24]	0.86	80%
		Emergency	2	400	0.86 [0.68, 1.04]	< 0.001	0%
		Both	4	504	0.13 [0.05, 0.22]	0.003	0%
	PPH	Elective	4	1319	0.60 [0.35, 1.05]	0.07	0%
		Emergency	1	180	0.15 [0.03, 0.68]	0.01	NA
		Both	3	288	0.52 [0.27, 1.02]	0.06	19%
	The need for additional uterotonics	Elective	7	1919	0.23 [0.09, 0.60]	0.003	86%
		Emergency	2	400	0.05 [0.00, 0.99]	0.05	93%
		Both	3	344	0.25 [0.06, 1.00]	0.05	54%
	The need for blood transfusion	Elective	7	1919	0.33 [0.15, 0.75]	0.008	3%
		Emergency	2	400	0.03 [0.00, 0.50]	0.01	NA
		Both	1	120	0.36 [0.11, 1.21]	0.10	NA

## Discussion

This meta-analysis confirmed that the amount of blood loss during the first 24 h after CD, the post-operative hemoglobin drop, the occurrence of PPH, the need for additional uterotonic agents and blood transfusion were significantly lower in women who received carbetocin during CD compared to women treated with oxytocin. The need for additional intervention was not significantly different between the two groups of women. While women received oxytocin experienced more headache than women who received carbetocin, all other adverse effects named nausea, vomiting, fever, abdominal pain and hypotension showed non statistical significant differences between the two groups.

The beneficial effect of carbetocin on blood loss was more evident in women who received 20 IU of oxytocin, under spinal and non-specified anesthesia, with twin pregnancy, obesity and general risk factors for PPH and in both elective and emergency CD. The improvement in hemoglobin drop was evident in women who received 30 and 40 IU of oxytocin, with twin pregnancy, hypertension, obesity and general risk factors for PPH and in both elective and emergency CD. The lower need for additional uterotonic agents were evident in all doses of oxytocin under all types of anesthesia with any risk factors and in both elective and emergency CD.

These advantages of carbetocin over oxytocin could be related to its prolonged half-life allowing prolonged more efficient uterine contractility and subsequent decrease in blood loss and other outcome parameters. The half-life of carbetocin was reported as 40 min compared to only 1–6 min half-life of oxytocin [10].

Another explanation may be related to the difficult suitable storage and transport environment for oxytocin in countries with limited resources. On evaluation of oxytocin samples in limited resource countries, 45.6% of the studied samples failed to pass the quality tests [13].

### Strengths and limitations

Our meta-analysis provides the premier available proof of the safety and efficacy of carbetocin compared to oxytocin in improving uterine contractions after CD with the subsequent decrease in post-operative blood loss, PPH, the need for additional uterotonic agents and the needs for blood transfusion without a significant increase in drug adverse effects. We included all the available published RCTs without any language limitations. Complete and careful data extraction, assessment of the risk of bias and full analysis (both quantitative and qualitative) was done for all included studies. Extensive subgroup analysis was done for all the reported outcomes for all variables including the dose of oxytocin, type of CD, type of anesthesia used and the risk factors for PPH. We also carefully assessed the quality of evidence for all outcomes.

The heterogeneity of the included studies regarding participants’ characteristics, doses and routes of oxytocin was the main limitation of our review. Not all articles reported the same outcomes. The location of research conduction was a mix of both developing and developed countries. Most of the studies were not prospectively registered. We tried to overcome these limitations through extensive subgroup analysis and performing the analysis using the random effect model.

### Comparison with existing reviews

A previous systematic review conducted by Wen and colleagues [29] in 2020. They included 17 RCT that used carbetocin during both VD and CD. There was no subgroup analysis or assessment for the quality of evidence.

Both Sun et al. [30] and Kalafat et al. [31] conducted a meta-analysis to compare carbetocin to oxytocin and included 24 and 30 studies, respectively. The included studies were a mix of benefits in both VD and CD. Both reviews lack subgroups analysis and assessment of quality of evidence. Beside this Kalafat review did not analyze the adverse effects of the drugs used.

Onwochei and colleagues [32] focused on women undergwent Cd but their trial included only 5 RCTs and this small number did not allow for any subgroup analysis.

A recent review by Jaffar and colleagues [33] included 46 trials. As it was a network meta-analysis, they compared carbetocin to various uterotonic agents not only oxytocin.

## Conclusion

This systematic review concluded that the use of carbetocin during CD is associated with less blood loss during 1st 24 h after the operation (high evidence), reduced the incidence of PPH (high evidence), the hemoglobin drop (moderate evidence), the need for additional uterotonic agents (high evidence), and blood transfusion (high evidence) when compared to oxytocin without increase in adverse effects.

We strongly recommend the use of carbetocin as a routine uterotonic agent in limited resource countries with unavailable storage and transport facilities to reach the goal of WHO of reducing maternal mortality based on its heat stable character. However being more expensive that oxytocin, individual cost effective assessment should be carried in each area guided by its resources to reach the decision of routine use or its use in high-risk women only.

## Supplementary Information

Below is the link to the electronic supplementary material.

Supplementary file1 (DOC 64 kb)

Supplementary file2 (DOCX 23 kb)

## Data Availability

No datasets were generated or analysed during the current study.
