# Supplementary material for: Carbetocin versus oxytocin in prevention of postpartum hemorrhage after cesarean delivery in high-risk women. A systematic review and meta-analysis
Source: Arch Gynecol Obstet. 2025 Apr 16;312(2):291–309. doi: 10.1007/s00404-025-08014-6 (PMC12334512; doi:10.1007/s00404-025-08014-6)
Supplement: Supplementary file 2 — Supplementary file2 (DOCX 23 kb) [file 404_2025_8014_MOESM2_ESM.docx]

Supplementary tables

Table S1 Search strategy

| Database | Keywords | Results |
| --- | --- | --- |
| Pubmed | (Carbetocin) [All Fields] AND (Caesarean [MeSH terms] AND (postpartum haemorrhage OR bleeding OR blood loss OR atony) [title/abstract] AND (randomized controlled trials OR randomized clinical trials OR randomized) [title/abstract] | 138 |
| Scopus | TITLE-ABS-KEY (carbetocin) AND TITLE-ABS-KEY (caesarean delivery OR caesarean section) AND TITLE-ABS-KEY (postpartum hemorrhage OR bleeding) | 173 |
| WOS | ts=postpartum hemorrhage OR postpartum bleeding OR PPH OR blood loss OR atony AND ts= carbetocin AND ts= Cesarean* | 165 |
| Clinical trial registration | Condition Postpartum hemorrhage  Other terms caesarean delivery  Intervention/treatment Carbetocin  Study status All studies | 44 |

Table S2 Excluded studies

| Study | Cause of exclusion |
| --- | --- |
| Akhter P, Pal SN, Begum S. Comparison between Carbetocin and Oxytocin in Active Management of 3rd Stage of Labour in Preventing Post Partum Hemorrhage. Mymensingh Med J. 2018 Oct;27(4):793-797. PMID: 30487496. | Vaginal delivery |
| Amornpetchakul P, Lertbunnaphong T, Boriboonhiransarn D, Leetheeragul J, Sirisomboon R, Jiraprasertwong R. Intravenous carbetocin versus intravenous oxytocin for preventing atonic postpartum hemorrhage after normal vaginal delivery in high-risk singleton pregnancies: a triple-blind randomized controlled trial. Arch Gynecol Obstet. 2018 Aug;298(2):319-327. | Vaginal delivery |
| Askar AA, Ismail MT, El-Ezz AA, Rabie NH. Carbetocin versus syntometrine in the management of third stage of labor following vaginal delivery. Arch Gynecol Obstet. 2011 Dec;284(6):1359-65. | Vaginal delivery |
| Boucher M, Nimrod CA, Tawagi GF, Meeker TA, Rennicks White RE, Varin J. Comparison of carbetocin and oxytocin for the prevention of postpartum hemorrhage following vaginal delivery:a double-blind randomized trial. J Obstet Gynaecol Can. 2004 May;26(5):481-8. | Vaginal delivery |
| Carrillo-Gaucín S, Torres-Gómez LG. Carbetocina y oxitocina: prevención de hemorragia posparto en pacientes con factores de riesgo para atonía uterina [Carbetocin and oxytocin: Prevention of postpartum hemorrhage in patients with risk factors for uterine atony]. Rev Med Inst Mex Seguro Soc. 2016;54 Suppl 3:S284-S290. Spanish. | Vaginal delivery |
| Cetin C, Tanoglu FB, Hanligil E, Gokce A, Pasin O, Ozcan P. Carbetocin versus oxytocin with or without tranexamic acid for prophylactic prevention of postpartum hemorrhage after a vaginal delivery: A randomized clinical trial. Gynecol Obstet Invest. 2023 Sep 30. | Vaginal delivery |
| Ghosh R, Owa O, Santos N, Butrick E, Piaggio G, Widmer M, Althabe F, Qureshi Z, Lumbiganon P, Katageri G, Walker D. Heat stable carbetocin or oxytocin for prevention of postpartum hemorrhage among women at risk: A secondary analysis of the CHAMPION trial. Int J Gynaecol Obstet. 2024 Jan;164(1):124-130. | Vaginal delivery |
| Leung SW, Ng PS, Wong WY, Cheung TH. A randomised trial of carbetocin versus syntometrine in the management of the third stage of labour. BJOG. 2006 Dec;113(12):1459-64. | Vaginal delivery |
| Liu, Hua; Xu, Xiu-Yun; Gu, Ning; Ye, Xiao-Dong; Wang, Zhi-Qun; Hu, Ya-Li; Dai, Yi-Min∗. Intravenous Administration of Carbetocin Versus Oxytocin for Preventing Postpartum Hemorrhage After Vaginal Delivery in High Risk Women: A Double-blind, Randomized Controlled Trial. Maternal-Fetal Medicine 2(2):p 72-79, April 2020. \| | Vaginal delivery |
| Maged AM, Hassan AM, Shehata NA. Carbetocin versus oxytocin for prevention of postpartum hemorrhage after vaginal delivery in high risk women. J Matern Fetal Neonatal Med. 2016;29(4):532-6. | Vaginal delivery |
| Monteo-Fenix, A. P., Vera, M. R., & Gorgonio, N. N.(2011).Double-blind randomized controlled trial comparing the effect of carbetocin and oxytocin for the prevention of postpartum hemorrhage among high risk women following vaginal delivery. Philippine Journal of Obstetrics and Gynecology, 35(4), 169-175 | Vaginal delivery |
| Nirmala K, Zainuddin AA, Ghani NA, Zulkifli S, Jamil MA. Carbetocin versus syntometrine in prevention of post-partum hemorrhage following vaginal delivery. J Obstet Gynaecol Res. 2009 Feb;35(1):48-54. | Vaginal delivery |
| Qiu 2014 Qiu Yi , Xie Han. The effect of carbetocin on preventing postpartum hemorrhage in vaginal delivery. Fudan University journal of medical sciences/ 2014. Vol 51 Issue 1: 102-105. | Vaginal delivery |
| Reyes OA, Gonzalez GM. Carbetocin versus oxytocin for prevention of postpartum hemorrhage in patients with severe preeclampsia: a double-blind randomized controlled trial. J Obstet Gynaecol Can. 2011 Nov;33(11):1099-1104. | Vaginal delivery |
| Samimi M, Imani-Harsini A, Abedzadeh-Kalahroudi M. Carbetocin vs. Syntometrine in Prevention of Postpartum Hemorrhage: a Double Blind Randomized Control Trial. Iran Red Crescent Med J. 2013 Sep;15(9):817-22. | Vaginal delivery |
| Su LL, Rauff M, Chan YH, Mohamad Suphan N, Lau TP, Biswas A, Chong YS. Carbetocin versus syntometrine for the third stage of labour following vaginal delivery--a double-blind randomised controlled trial. BJOG. 2009 Oct;116(11):1461-6. | Vaginal delivery |
| van der Nelson H, O'Brien S, Burnard S, Mayer M, Alvarez M, Knowlden J, Winter C, Dailami N, Marques E, Burden C, Siassakos D, Draycott T. Intramuscular oxytocin versus Syntometrine^®^ versus carbetocin for prevention of primary postpartum haemorrhage after vaginal birth: a randomised double-blinded clinical trial of effectiveness, side effects and quality of life. BJOG. 2021 Jun;128(7):1236-1246. | Vaginal delivery |
| Vernekar SS, Goudar SS, Metgud M, Pujar YV, Somannavar MS, Piaggio G, Carvalho JFDE, Revankar A, Althabe F, Widmer M, Gulmezoglu AM, Goudar SS. Effect of heat stable carbetocin vs oxytocin for preventing postpartum haemorrhage on post delivery hemoglobin-a randomized controlled trial. J Matern Fetal Neonatal Med. 2022 Dec;35(25):8744-8751. | Vaginal delivery |
| Widmer M, Piaggio G, Nguyen TMH, Osoti A, Owa OO, Misra S, Coomarasamy A, Abdel-Aleem H, Mallapur AA, Qureshi Z, Lumbiganon P, Patel AB, Carroli G, Fawole B, Goudar SS, Pujar YV, Neilson J, Hofmeyr GJ, Su LL, Ferreira de Carvalho J, Pandey U, Mugerwa K, Shiragur SS, Byamugisha J, Giordano D, Gülmezoglu AM; WHO CHAMPION Trial Group. Heat-Stable Carbetocin versus Oxytocin to Prevent Hemorrhage after Vaginal Birth. N Engl J Med. 2018 Aug 23;379(8):743-752. | Vaginal delivery |
| Terblanche NC, Sharman JE, Jones MA, Gregory K, Sturgess DJ. Uterine atony prophylaxis with carbetocin versus oxytocin and the risk of major haemorrhage during caesarean section: A retrospective cohort study. *Anaesth Intensive Care*. 2023;51(4):288-295. | Non RCT |
| Luni Y, Borakati A, Matah A, Skeats K, Eedarapalli P. A prospective cohort study evaluating the cost-effectiveness of carbetocin for prevention of postpartum haemorrhage in caesarean sections. *J Obstet Gynaecol*. 2017;37(5):601-604. | Non RCT |
| Nucci B, Aya A, Aubry E, Ripart J. Carbetocin for prevention of postcesarean hemorrhage in women with severe preeclampsia: a before-after cohort comparison with oxytocin. *J Clin Anesth*. 2016;35:321-325. | Non RCT |
| Delorme P, Kayem G, Legardeur H, et al. Carbetocin versus Oxytocin for the Prevention of Postpartum Hemorrhage in Cesarean Deliveries: A Retrospective Study of Two Consecutive Periods. *AJP Rep*. 2020;10(3):e241-e246. | Non RCT |
